# Lithium-ion Battery Thermal Safety by Early Internal Detection, Prediction and Prevention

**DOI:** 10.1038/s41598-019-49616-w

**Published:** 2019-09-13

**Authors:** Bing Li, Mihit H. Parekh, Ryan A. Adams, Thomas E. Adams, Corey T. Love, Vilas G. Pol, Vikas Tomar

**Affiliations:** 1 School of Aeronautics and Astronautics, Purdue University, West Lafayette, IN 47907 USA; 2 Davidson School of Chemical Engineering, Purdue University, West Lafayette, IN 47907 USA; 3 Naval Surface Warfare Center, Crane Division, Crane, IN 47522 USA; 4 U.S. Naval Research Laboratory, Washington, DC 20375 USA

**Keywords:** Batteries, Chemical engineering

## Abstract

Temperature rise in Lithium-ion batteries (LIBs) due to solid electrolyte interfaces breakdown, uncontrollable exothermic reactions in electrodes and Joule heating can result in the catastrophic failures such as thermal runaway, which is calling for reliable real-time electrode temperature monitoring. Here, we present a customized LIB setup developed for early detection of electrode temperature rise during simulated thermal runaway tests incorporating a modern additive manufacturing-supported resistance temperature detector (RTD). An advanced RTD is embedded in a 3D printed polymeric substrate and placed behind the electrode current collector of CR2032 coin cells that can sustain harsh electrochemical operational environments (acidic electrolyte without Redox, short-circuiting, leakage etc.) without participating in electrochemical reactions. The internal RTD measured an average 5.8 °C higher temperature inside the cells than the external RTD with almost 10 times faster detection ability, prohibiting thermal runaway events without interfering in the LIBs’ operation. A temperature prediction model is developed to forecast battery surface temperature rise stemming from measured internal and external RTD temperature signatures.

## Introduction

Lithium-ion batteries (LIBs) have a profound impact on the modern industry and they are applied extensively in aircraft, electric vehicles, portable electronic devices, robotics, etc.^1–3^. However, LIBs are prone to failure due to overheating, over-discharging, overcharging or short-circuit^4^. During such abusive events, chemical energy quickly converts to thermal energy, which leads to heat accumulation, then thermal runaway, fire and explosion^5^. Although there are various safety installations at present such as pressure burst discs, shutdown separators, and one-shot fuses, severe battery hazards have been reported repeatedly^4^. This calls for an in-service thermal monitoring approach. One simple way applied intensively is to measure battery surface temperature^6,7^. Despite the simplicity, external surface temperature monitoring has been shown ineffective due to dissipated heat for LIB safety management^8^. Temperature difference within LIB during battery failure impairs reliability and efficiency of surface temperature based safety management. Finegan *et al*. analyzed short-circuit condition of 18650 LIB cells with infrared camera and the temperature rise occurred asynchronously across the cell^9^. Liu *et al*. and Zhao *et al*. simulated temperature evolution during short circuit of LIB with finite element method, and they observed significant temperature gradient in both thickness and height direction of shorted Li-ion pouch cells^10,11^. A comparison of electrode and battery surface temperature showed that the external surface-based measurement detected peak temperature with reduced magnitude and time delay, and electrode temperature should be the key for prevention of thermal runaway^8^. Zhang *et al*. introduced internal short-circuit to LIB with memory alloy, and it took 18 s on average for battery surface temperature to reach peak value after short circuit was triggered^12^. For real-time electrode temperature monitoring, Zhang *et al*. used a T-type thermocouple inserted *between* the anode and separator of 18650 LIB^8^. Electrode temperature during battery short-circuit was recorded and compared with battery surface temperature. Novais *et al*. inserted a fiber bragg grating sensor between double layer separators of the pouch cell to measure temperature change *in-operando*^13^. During cycling, fluctuation in the electrode temperature was reported.

Such internal sensor-based electrode temperature measurements have offered superior temperature measurement efficiency and accuracy. It has also been applied with widely adopted short circuit tests for LIB safety analysis, where the shorted battery are subjected to risk of thermal runaway, fire and explosion^14,15^. When the examined LIBs were shorted to simulate thermal runaway conditions, temperature differences up to 50 °C were observed between the internal and external thermocouples, and the internal thermocouple reported the peak temperature nearly 20 s in advance^8^. However, it was noted that the sensor embedded between the cathode and anode^8,13^ may impede electrochemical reaction during the battery operation. It is challenging to maintain the contact between porous electrode material and a sensor without damaging the electrode, as microcracks are prone to form in porous structure under compression and lead to material fracture^16^. During battery thermal hazards such as a thermal runaway phenomena, violent temperature rise leads to cracking of the electrode material^17^ and other particle based structures^18^, which can impair the contact between the sensor and electrode material. Our previous work also showed that direct mechanical load influences the electrochemical performance of LiCoO_2_ (LCO) cathode significantly^19^, implying that inserting a sensor between the electrodes may not be preferable.

In this work, a novel method for incorporating a resistance temperature detector (RTD) behind the cathode current collector of a LIB via additive manufacturing was developed for electrode damage minimization and internal LIB *in-operando* temperature measurement efficiency improvement. Customized LIBs (CR2032 coin cells) were tested for structural and electrochemical stability in vibrational loading environments. The thermal hazard detecting capability was evaluated using intentional heat rise due to an applied external short circuit^8^. Internal RTD placement yielded significantly superior measuring efficiency and accuracy in comparison to literature reports.

## Results

### RTD sensor embedded lithium-ion coin cell for electrode temperature measurement

**Figure 1:**
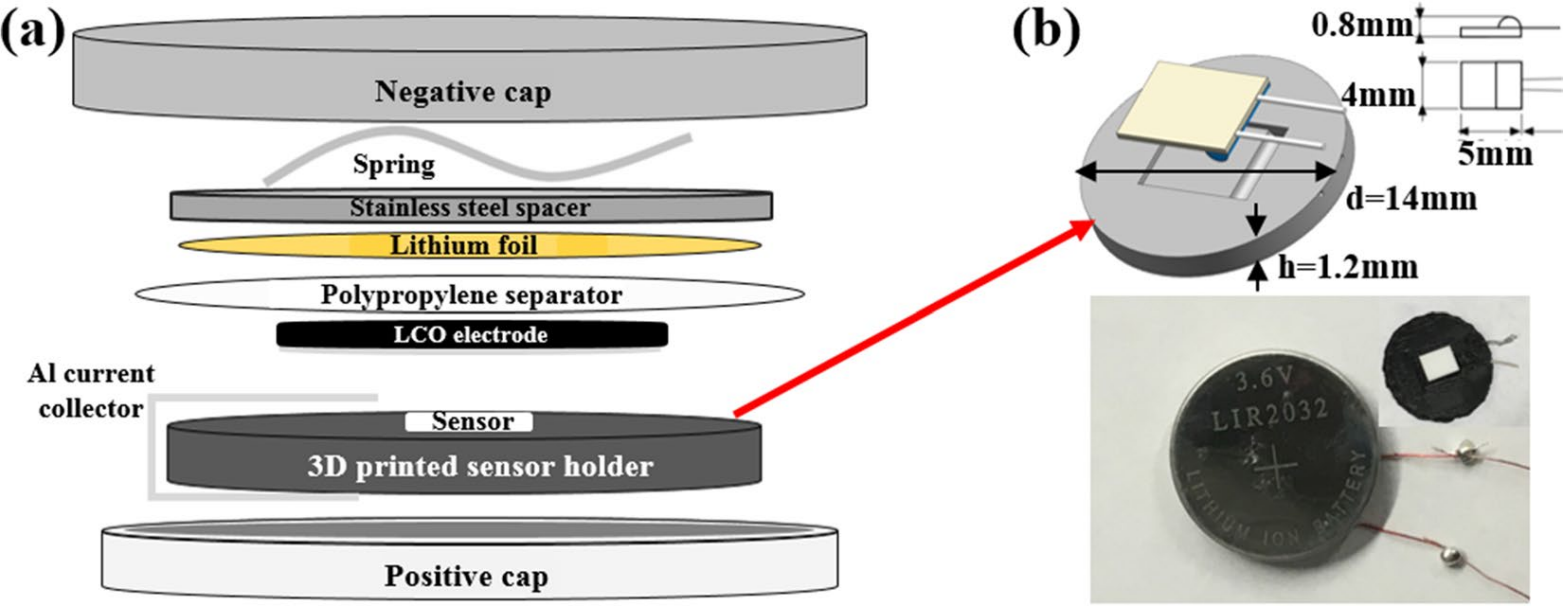
(**a**) Schematic of customized RTD embedded LIB coin cell; (**b**) RTD embedded PLA spacer and CR2032 cell with internal RTD. Dimension of the RTD embedded spacer was comparable to ordinary CR2032 coin cell spacer, allowing for reliable sensor-electrode contact and cell sealing after assembly.

For the CR2032 coin cells employed in this work, the RTD was incorporated into a customized polylactic acid (PLA) spacer with additive manufacturing, which was placed beneath the cathode as shown in Fig. 1. Sensor placement on the anode side is being addressed in a separate work. Temperature measuring efficiency and accuracy as well as electrochemical stability of customized spacer were analyzed and details of these analyses are provided in the Method section.

**Figure 2:**
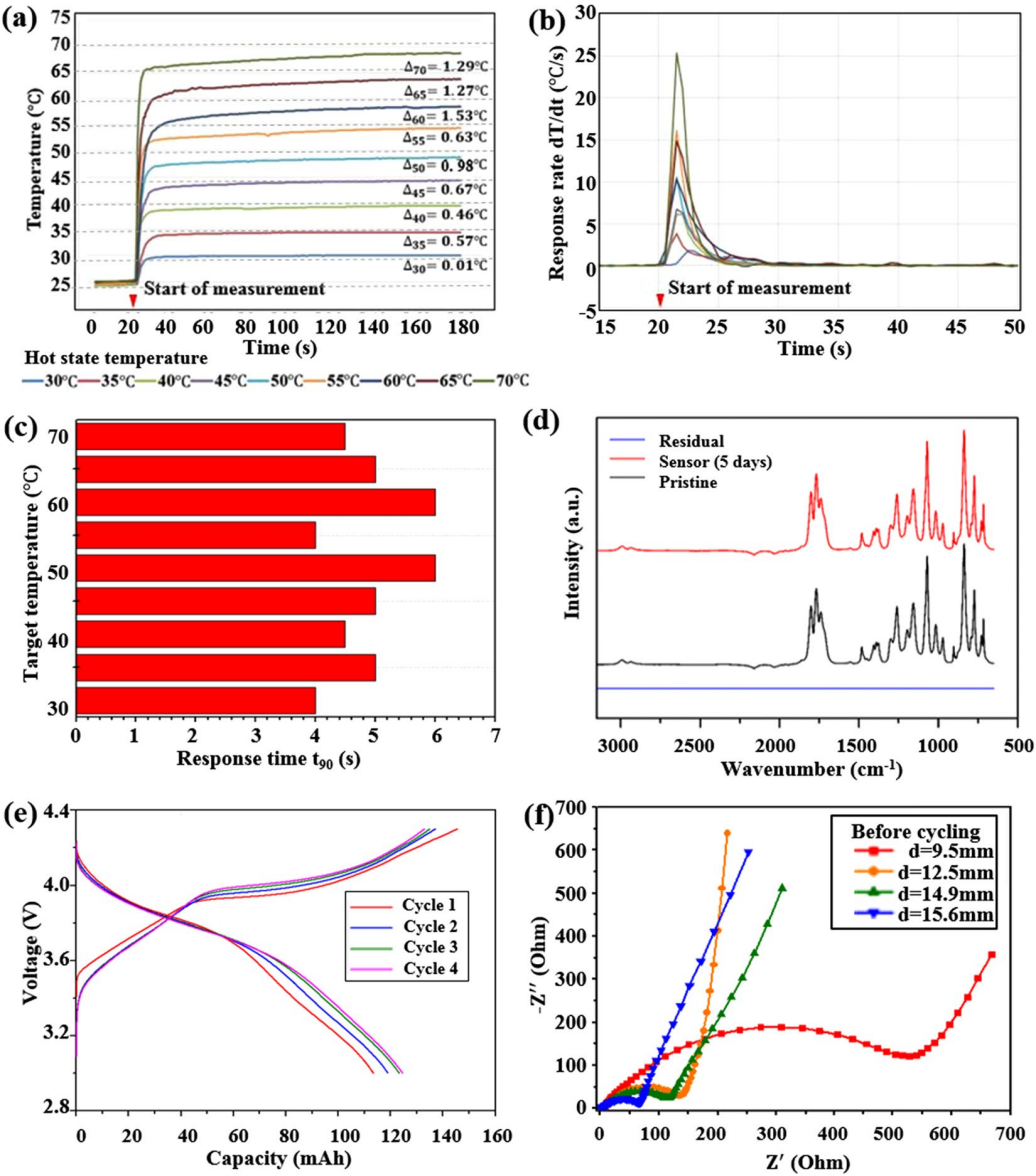
(**a**) Hot stage temperature measurement with RTD embedded spacers; (**b**) RTD embedded spacer measurement response rate; (**c)** RTD embedded spacer measurement response time t_90_; (**d**) FTIR spectra for electrolyte stability testing; (**e**) Comparison of charge/discharge curves for sensor embedded cell over vibration; (**f**) Electrochemical impedance spectroscopy of the constructed cells at OCV (3.1 V vs Li/Li+) with RTD.

The result of hot stage temperature measurement with RTD embedded spacer is presented in Fig. 2a, where the RTD embedded spacers were clamped onto the hot stage at t = 20 s. As shown, the customized spacer provides temperature readings with an error <1 °C up to 55 °C, and an average error of 0.82 °C. The response rate of the spacer is evaluated in Figs. 2b,c, where the response time t_90_ is defined as the time required for RTD to capture 90% of the total temperature shift^20^. The average value of t_90_ is 5 s for the RTD embedded spacer, which agrees with the observation for the RTD response rate. The t_90_ also presents no dependency on target temperature within the assessed temperature range. These results indicate that the RTD embedded spacer could detect thermal hazards with high efficiency and has limited measuring error over the temperature range covering room temperature to the onset temperature of LIB thermal runaway^21^.

In Fourier-transform infrared (FTIR) spectral analysis of RTD embedded spacer inertness, there are no changes in band intensity and frequency of the electrolyte as shown in Fig. 2d. The consistent FTIR spectra indicate that there is no transition in electrolyte composition and concentration^22^, and the RTD embedded spacer is electrochemically inert, avoiding interference to LIB operation. During cycling of the RTD embedded cell, based on the NAVSEA 9310 vibration test specifications, the LIB cell presents consistent charge/discharge behavior and the cell structure is robust under applied vibrational load.

Customized CR2032 coin cells with 12.5 mm diameter electrodes were prepared as discussed previously. Cells were cycled between 3.0 V and 4.3 V (Fig. 2e) using “C/12 rate”. Cells report a discharge capacity of about 120 mAh g^−1^ and a charge capacity of about 140 mAh g^−1^ as shown in Fig. 2e. First cycle capacity of the cell is slightly lower due to the formation of a passivation layer on the surface of the cathode^23^. Following that, the charge and discharge profiles overlapped well for the next cycles. During charging, delithiation starts at around 3.9 V; while discharging, lithiation of the cathode starts at around 3.7 V without altering the profile of LCO because of the sensor assembly.

Electrochemical impedance spectroscopy (EIS) of the customized CR2032 cells with various cathode diameter (9.5 mm, 12.5 mm, 14.9 mm, 15.6 mm) was conducted at the OCV potential (Fig. 2f). In the high-medium frequency area, the plot depicts depressed semicircles and in the high-frequency area it shows linear Warburg impedance. Ohmic resistances for all the electrodes were similar to each other, ~2.5 Ω. Charge transfer resistance differed from each other. An inverse relationship between charge transfer resistance and the size of the electrodes was observed. For the smallest electrode, charge transfer impedance was 527 Ω, whereas for the largest electrode, it was 65 Ω. The difference in charge transfer resistance between the smallest and largest electrodes arose because the effective area became a significant factor in regions of low frequency, as more charge could pass through the large area, causing impedance to decrease^24^. High charge transfer resistance has also been previously reported in coin cells with limited dimension^25^. The impedance of RTD embedded cells was found to be comparable with the other CR2032 coin cells fabricated in a controlled lab environment^25,26^, and the contribution of the customized spacer with an RTD to the cell impedance was limited. Since all four coin cells had limited and comparable Ohmic resistance, the effect of 3D printed spacer on cell performance was negligible and the results obtained from short circuit test of RTD embedded cells could be employed for safety management of ordinary coin cells.

### External short circuit test and real-time electrode temperature monitoring

**Figure 3:**
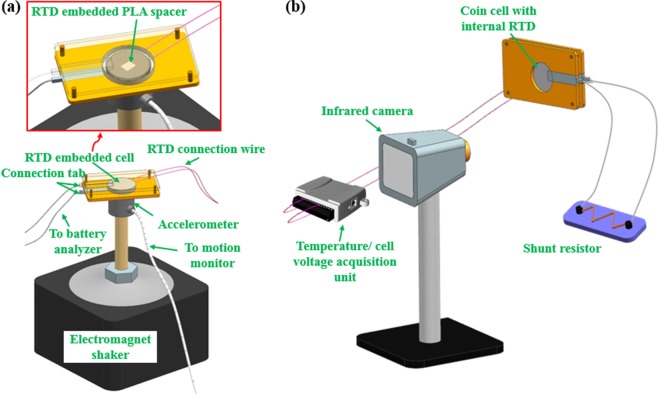
(**a**) Portable battery cycling setup for LIB performance analysis during vibration; (**b**) LIB short circuit testing and temperature monitoring platform.

Short circuits are a common concern for aged batteries due to dendrite formation and separator degradation^27–29^, and it is also common in transportation-related accidents such as electric vehicle crashes^30,31^. Short circuits can induce dramatic changes in electrode structure and the electrochemical environment of the battery^11^. An external short circuit test was employed for evaluation of thermal hazard capturing capability of the RTD embedded cells in this work. The platform for external short circuit test and temperature monitoring is shown in Fig. 3b with a detailed testing procedure provided in the Method section.

The electrode and battery surface temperature were recorded for the first hour as short circuit related electrochemical reactions were observed to be negligible afterward. An infrared camera (FLIR E40) was used for battery surface temperature recording as a comparison tool for the external RTD measurement result.

**Figure 4:**
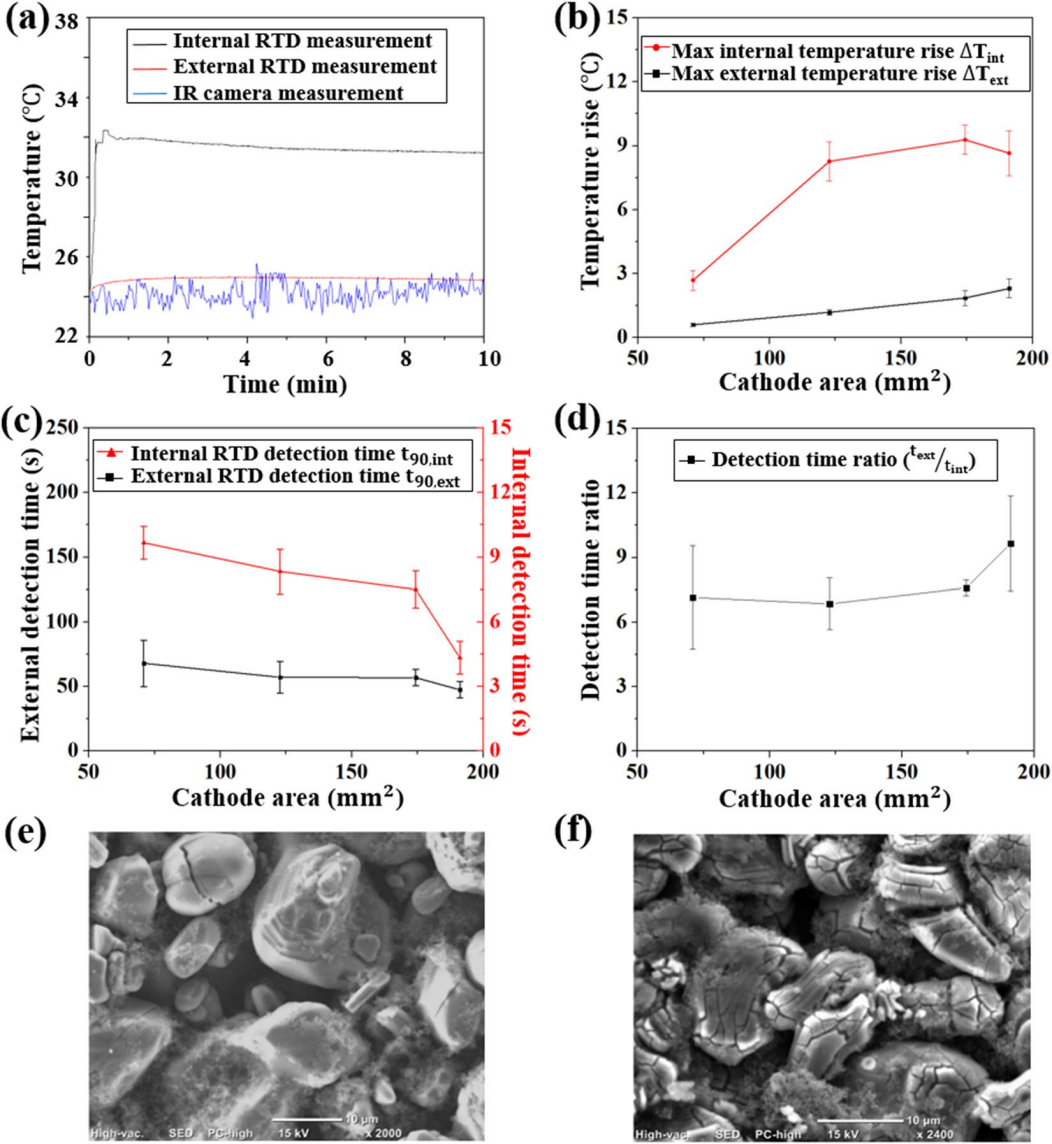
(**a**) Temperature measurements with internal RTD, external RTD and infrared camera in the short circuit test; (**b**) Maximum temperature rise detected by internal and external RTDs; (**c**) RTD detection time t_90, int_ and t_90, ext_ of internal and external RTDs; (**d**) Detection time ratio t_ext_/t_int_; (**e**) SEM image of LCO cathode before short circuit test and (**f**) after short circuit test.

A comparison of examined LIB temperature profiles obtained with internal RTD, external RTD, and infrared camera over the first 10 mins of the short circuit test (cathode diameter: 12.5 mm) is shown in Fig. 4a. The temperature obtained with the infrared camera is unreliable due to the high-level fluctuation which mainly originates from changes in ambient convection characteristics^32^. In the comparison of RTD measurements, the first difference is in the maximum temperature: T_max_ captured by internal RTD is on average 5.8 °C higher than the external RTD measured average (Fig. 4b). This difference contributes to the thermal contact resistance, which is most significant at electrode-separator contact surface and battery poles^33,34^. The internal RTD measurement avoids the high-level temperature gradient, thus providing accurate electrode temperature monitoring for thermal event detection. The second main difference is in the peak temperature detection time: external RTD detects peak temperature when the internal RTD reading is stabilized or starts decreasing. The measuring efficiency difference arises from energy loss in heat conduction: when heat generated in the electrodes is conducted to the battery surface, part of it is consumed by the temperature rise of battery components, and part of it is dissipated to the air. As a result, the external RTD will fail to reflect the actual temperature rising rate, leaving the cell continuously exposed to potential thermal hazards.

For further evaluation of measurement efficiency, detection time t_90_ is compared for internal and external RTDs. As discussed, t_90_ is defined as the time to detect 90% of the maximum temperature rise measured by external RTD (ΔT_ext_). ΔT_ext_ is compared with the maximum temperature rise measured by the internal RTD (ΔT_int_) in Fig. 4(b) and with the t_90_ of two RTDs in Fig. 4c. The internal RTD detects 90% of ΔT_EMax_ in 7.45 s on average, which is 7–10 times faster than the external RTD. The measuring efficiency difference builds up with the increase of cathode mass as shown in Fig. 4d, indicating that internal sensor based measurement will be more instructive for thermal hazard detection in LIBs with higher capacity. The high measuring efficiency of the internal RTD is attributed to the well maintained sensor-electrode contact. Reliable sensor-electrode contact is maintained by the pressure load applied during cell fabrication, allowing for effective heat conduction from the current collector to internal RTD. In existing work on electrode temperature measurement, the sensors were directly applied onto the porous electrode material^8^ with limited control of sensor-electrode contact and installation stress. Excessive installation pressure could lead to electrode material damage and impair battery performance^35^. Also, electrode particle cracking and peel-off occurred during LIB thermal runaway, which further impairs the unsecured sensor-electrode contact and can be observed in SEM images acquired on the cathode before and after the short circuit test, as shown in Figs. 4e,f.

**Figure 5:**
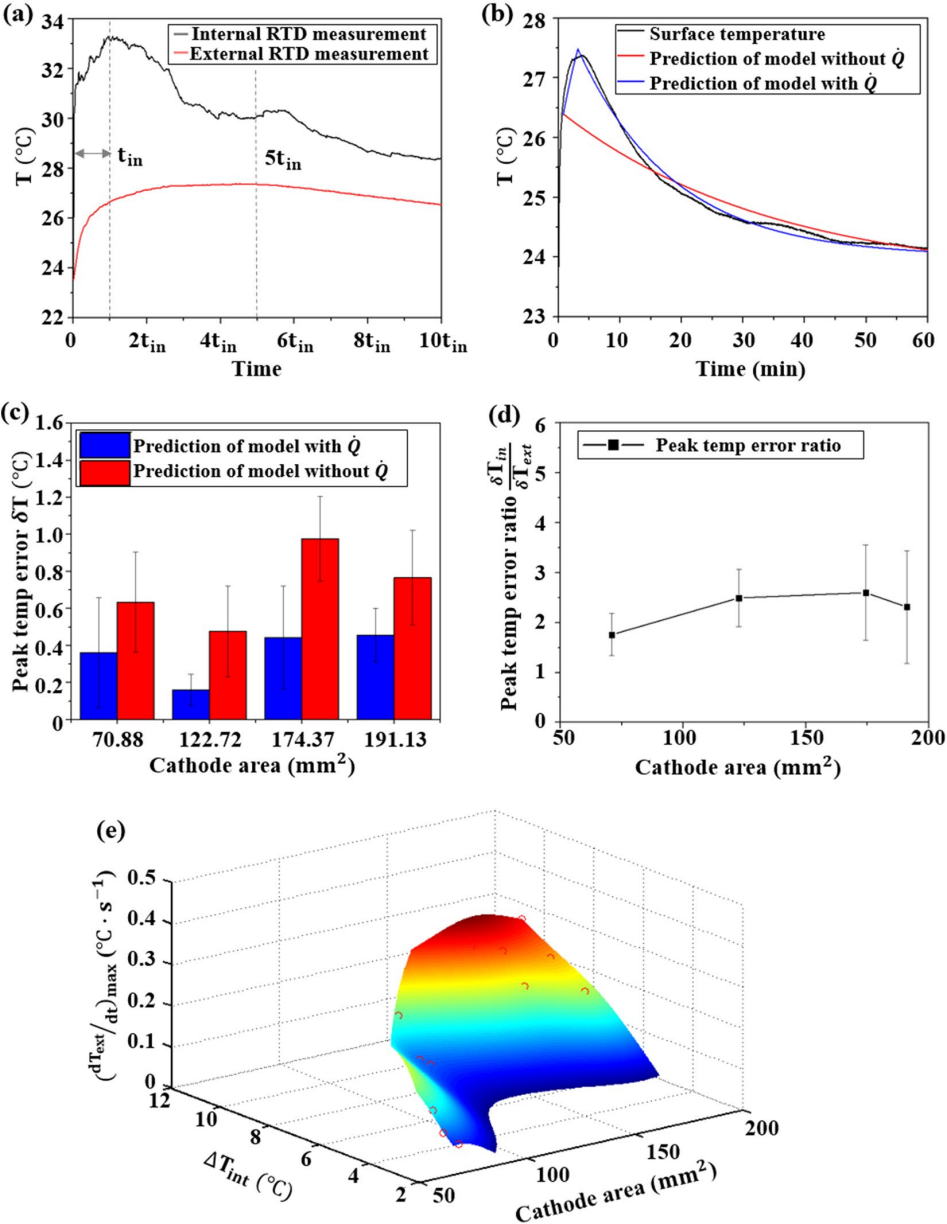
(**a**) Two phases of heat transfer in battery short circuit test; (**b**) Representative comparison of temperature model predictions; (**c**) Peak temperature prediction error comparison; (**d**) Peak temperature prediction error ratio; (**e**) Relation between cathode mass, peak internal temperature and maximum battery surface temperature rising rate.

Despite electrode temperature monitoring with improved efficiency and accuracy, an internal RTD can also be used for the prediction of cell temperature evolution after the detected thermal hazard. When high temperature is detected in a LIB pack, involved cells will be removed from the circuit^36^ and the subsequent cooling process is typically accomplished by convection in air^32^. As the current drains rapidly in the short circuit test (within 5 s), the temperature decrease process of CR 2023 coin cells can be modeled with natural convection, and a general energy balance equation of LIB cells can be written as^32^:

1 $${\rm{mC}}\frac{{{\rm{dT}}}_{{\rm{ext}}}}{{\rm{dt}}}=\dot{{\rm{Q}}}-{\rm{hA}}({{\rm{T}}}_{{\rm{ext}}}-{{\rm{T}}}_{\infty })$$

where m is cell mass, C is heat capacity of the cell, h is convective heat transfer coefficient, A is convection area, ${{\rm{T}}}_{\infty }$ is ambient temperature, T_ext_ is battery surface temperature and $\dot{{\rm{Q}}}$ is the internal heat source term. A simple way to obtain the analytical solution of Eq. (1) is to assume uniform temperature distribution of the cell and neglect the $\dot{{\rm{Q}}}$ term during the cooling process, which provides a solution for T_ext_ as^32^:

2 $$({{\rm{T}}}_{{\rm{ext}}}-{{\rm{T}}}_{\infty })=({{\rm{T}}}_{{\rm{e}}0}-{{\rm{T}}}_{\infty })\exp (-\frac{{\rm{hA}}}{{\rm{mc}}}{\rm{t}})=({{\rm{T}}}_{{\rm{e}}0}-{{\rm{T}}}_{\infty })\exp (-\frac{{\rm{t}}}{{\rm{\tau }}})$$

with T_ext_ = T_e0_ at the beginning of convective cooling and τ is the convection time constant ($\frac{{\rm{hA}}}{{\rm{mc}}}$) that can be determined with temperature evolution in natural convection cooling of the cell. However, our previous measurements show that there is a significant temperature gradient within the cell, and the thermal conduction from the electrode to the cell surface is non-negligible at the start of the cooling process. Thus, the model in Eq. (2) will fail to reflect the actual change of battery surface temperature and it is necessary to consider the thermal energy transferred from the electrode for accurate surface temperature prediction. Considering this, the cooling process is separated into two phases: in the first time period the electrode temperature is different from the cell surface temperature, and the electrode system provides the internal heat source term $\dot{{\rm{Q}}}$, in the second phase cell temperature is relatively uniform and $\dot{{\rm{Q}}}$ can be neglected. Based on the measurement efficiency comparison of internal and external RTD in Fig. 4(d), separation for periods 1 and 2 can be set at 5 t_in_, where t_in_ is the time for the internal RTD to detect the maximum electrode temperature, as shown in Fig. 5(a). The new energy balance equation for the battery can be written as:

3a $$\{{\rm{mC}}\frac{{{\rm{dT}}}_{{\rm{ext}}}}{{\rm{dt}}}=\dot{{\rm{Q}}}-{\rm{hA}}({{\rm{T}}}_{{\rm{ext}}}-{{\rm{T}}}_{\infty }){{\rm{t}}}_{{\rm{in}}}\le {\rm{t}} < 5{{\rm{t}}}_{{\rm{in}}}$$

3b $$\{{\rm{mC}}\frac{{{\rm{dT}}}_{{\rm{ext}}}}{{\rm{dt}}}=-{\rm{hA}}({{\rm{T}}}_{{\rm{ext}}}-{{\rm{T}}}_{\infty })5{{\rm{t}}}_{{\rm{in}}}\le {\rm{t}}$$

The internal heat source term $\dot{{\rm{Q}}}$ is given as:

4 $$\dot{{\rm{Q}}}=-\,{{\rm{m}}}_{{\rm{e}}}{{\rm{C}}}_{{\rm{e}}}\frac{{{\rm{dT}}}_{{\rm{in}}}}{{\rm{dt}}}$$

where T_in_ is the temperature obtained with internal RTD, m_e_ and C_e_ are the mass and heat capacity of the system that possesses a temperature of T_in_. It is assumed that for the LiCoO_2_ cathode, the Li anode and stainless-steel spacer above the anode possess a temperature of T_in_, considering the tight contact between these layers, and the values of m_e_ and C_e_ are calculated, accordingly^37,38^. After reaching the maximum value at t_in_, T_in_ is modeled to decay exponentially:

5 $${{\rm{T}}}_{{\rm{in}}}={{\rm{T}}}_{\infty }+({{\rm{T}}}_{{\rm{i}}0}-{{\rm{T}}}_{\infty })\cdot \exp (\,-\,{\rm{a}}\cdot {\rm{t}})$$

where a is the time coefficient to be determined and the value of T_in_ over t_in_ ≤ t ≤ 2t_in_ is used for the derivation of a. Then the solution of Eq. (3a) can be obtained numerically with MATLAB providing the initial value of T_ext_ at *t* = t_in_, and the solution for Eq. (3b) is provided by Eq. (2). A comparison of battery surface temperature predictions with and without the contribution of the internal heat source $\dot{{\rm{Q}}}$ is shown in Fig. 5(b). The new model reduces error in maximum surface temperature prediction as shown in Fig. 5c. The ratio of T_EMax_ prediction error remains steady over different cathode sizes and the model can be applied to cells with higher electrode mass. The proposed model can also predict battery temperature evolution after thermal hazard detection and avoid thermal hazard after removal of the cell.

The relation between temperatures measured with internal and external RTDs can also be used for improving the efficiency of surface temperature-based battery thermal runaway detection. A curved surface correlating maximum surface temperature increasing rate with cathode area, *A*, and cathode temperature increase, Δ*T*_*int*_, in short circuit is plotted by biharmonic spline interpolation and 4^th^ order polynomial fitting with MATLAB in Fig. 5e. The relation can be written as:

6 $$\begin{array}{rcl}{(\frac{d{T}_{ext}}{dt})}_{max} & = & -1.83+0.060\cdot A+0.36\cdot \Delta {T}_{int}-6.88\cdot {10}^{-4}\cdot {A}^{2}-7.17\cdot {10}^{-3}\\  &  & \cdot A\cdot \Delta {T}_{int}-0.011\cdot \Delta {T}_{int}^{2}+3.28\cdot {10}^{-6}\cdot {A}^{3}+4.50\cdot {10}^{-5}\cdot {A}^{2}\\  &  & \cdot \Delta {T}_{int}+2.14\cdot {10}^{-4}\cdot A\cdot \Delta {T}_{int}^{2}-5.77\cdot {10}^{-9}\cdot {A}^{4}-7.84\cdot {10}^{-8}\\  &  & \cdot {A}^{3}\cdot \Delta {T}_{int}-8.83\cdot {10}^{-7}\cdot {A}^{2}\cdot \Delta {T}_{int}^{2}\end{array}$$

The relation between Δ*T*_*int*_ and cathode area, *A*, of a coin cell is fitted with a cubic polynomial function:

7 $$\Delta {T}_{int}=-\,21.364+3.874\cdot A-0.164\cdot {A}^{2}+2.301\cdot {10}^{-3}\cdot {A}^{3}$$

**Figure 6:**
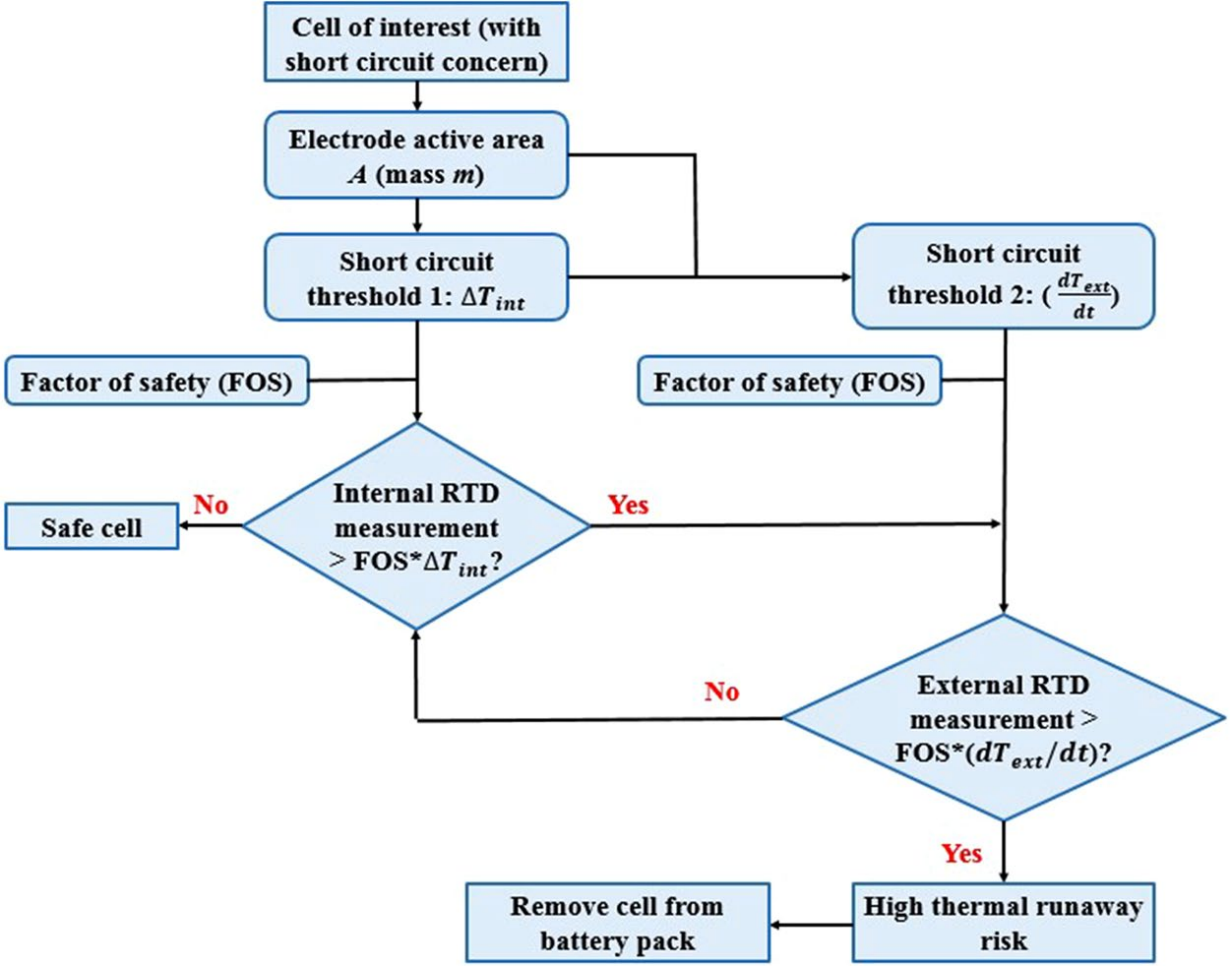
Strategy for protecting coin cell against short circuit related thermal runaway.

It is found from Eq. (6) that the increasing rate of battery surface temperature in short circuit related thermal runaway is dependent on both electrode temperature rise and electrode mass. This increase arises because exothermic reaction in short circuit is mainly composed of SEI decomposition, reaction between cathode, anode and electrolyte and electrolyte decomposition at the electrolyte-electrode interface [1], and these reactions are dependent on electrochemically active mass as shown in Eq. (7). Electrode surface area determines the rate of thermal energy transfer from the electrode to battery surface. Thus, the thermal runaway risk level cannot be simply predicted with the change in surface temperature rise, but the cell capacity also needs to be taken into consideration. For CR 2032 cell with specific LCO cathode mass, when the increasing rate of surface temperature approaches the top part of the curved surface in Fig. 6e, there will be a high risk of thermal runaway and effective cooling such as forced air cooling should be applied to control the electrode temperature and detrimental thermal gradient across the cell^39^. For similar increasing rate of surface temperature, batteries with lower electrode mass will be more prone to thermal hazards, and a cell capacity dependent safety temperature threshold can be determined based on external RTD measurement with the relation between internal and external RTD reading established. Besides, some fluctuation was observed in temperature measured by internal RTD during the short circuit event. It represented instability in local heat generation and transfer. The fluctuation originated from changes in LIB structure during short circuit, including lithium dendrite formation, current collector dissolution, electrode particle delamination, gas generation, etc. Dendrite could reduce cell resistance and trigger internal shorting^27^, which could enhance the short circuit. Current collector dissolution and gas generation influenced cell impedance and local heat transfer condition^40,41^. Electrode particle could delaminate during thermal runaway^42^, leading to abrupt drop in cell capacity and heat generation rate at the region of delamination. Current collector dissolution and electrode particle delamination observed in short circuit test can be found in Supplementary Fig. S1. The local measurement capability of internal RTD captured these transient regional processes, and it was instructive for LIB thermal runaway detection and prevention.

For prevention of short circuit related thermal runaway, a thermal runaway risk assessing strategy is developed for CR2032 coin cell based on internal and external RTD measurements as well as established relations between the electrode and battery surface temperature from Eqs (6 and 7):

This thermal runaway prevention strategy consists of internal RTD and external RTD based protection. Due to the superior measuring efficiency, internal RTD measurement is critical when short circuit is of concern for a cell. Electrode temperature rise, Δ*T*_*int*_, is used as the early signature of thermal runaway and if the measured value excesses range for safe battery operation, the increasing rate of battery surface temperature will be calculated with the external RTD and employed for verification of assessment. If the battery surface temperature is rising in an unsafe manner based on Eq. (6), a high risk for thermal runaway is identified for the analyzed cell and it should be disconnected immediately. Scientific experimental efforts are underway to test multiple RTD sensors in pouch full-cell configurations for local heat generation mapping of the electrode.

## Conclusion

In this study, a RTD sensor embedded PLA spacer was developed and incorporated inside the CR2032 coin cells for *in-operando* temperature monitoring. The customized cell presented reliable sensor-electrode contact and high structural robustness during cycling over continuous vibration, based on U.S. Navy standards. In the short circuit test, the internal RTD provided superior performance in measuring the electrode temperature compared to the external RTD. In addition, the internal RTD reported peak temperature 7–10 times faster than the external RTD, providing a better chance for battery hazard prevention. Electrode temperature measurement with an internal RTD was also employed for battery surface temperature prediction in short circuit, providing surface temperature evolution prediction with reduced error. Finally, the relation between internal RTD measurement, external RTD measurement and electrode mass was obtained for reliable short circuit detection and prediction based on battery surface temperature measurement.

## Methods

### RTD sensor embedded lithium-ion coin cell fabrication

To develop a feasible approach to detect battery thermal runaway *in-operando* and meet requirement on commercial LIBs, the design of a customized RTD embedded LIB cell was dictated by three key factors: (1) to acquire the cathode electrode temperature accurately and effectively; (2) to eliminate sensor induced interference due to LIB operation; and (3) to minimize sensor induced electrode damage. For accurate real-time electrode temperature monitoring, reliable sensor-electrode contact is desired, which requires that the sensor surface pairs well with the electrode. Pt1000 RTD (by Omega Engineering Inc.) with a 4 mm × 5 mm flat Al_2_O_3_ sensing surface was selected in this work. Pt1000 RTD has been extensively applied in process temperature monitoring, including LIB electrolyte stability analysis^43,44^, and LIB cycling^45^. The Pt sensing element employed in this work had a temperature dependent resistance, R, of:

8 $${\rm{R}}=(1+3.9083\cdot {10}^{-3\circ} {\rm{C}}^{-1}\cdot {\rm{T}}-{5.77510}^{-7 \circ} {\rm{C}}^{-2}\cdot {\rm{T}}^{2}-{4.18310}^{-12 \circ} {\rm{C}}^{-4}\cdot {\rm{a}}\cdot {\rm{T}}^{3}){\rm{k}}\Omega $$

where a = (T-100) °C for T < 0 °C and a = 0 °C for T > 0 °C.

The RTD provided an average sensitivity of 3.883 Ω/°C within the ordinary battery operation temperature of −10 °C to 50 °C^46^.

A strip of aluminum current collector was applied across the PLA spacer for cell conductivity. By embedding the customized spacer in the CR2032 cell, a new pattern of measuring electrode temperature from the electrode current collector was achieved, which eliminated sensor induced disturbance to battery operation by removing the sensor from the gap between the electrodes. Thickness variation of the RTD embedded spacer was controlled within 10 μm for contact reliability between the RTD and the electrode. The well-maintained spacer-electrode pairing can improve measurement efficiency and control electrode damage during temperature monitoring. In addition to application in CR 2032 coin cell shown in Fig. 1, 3D printing technique provided visibility for the RTD embedded spacer to be applied in commercial LIBs, including 18650 cell. With customized geometry design, the RTD can be applied to measure electrode temperature at different locations of the jelly roll. Illustration of RTD application in 18650 cell is provided in Supplementary Fig. S2.

### RTD embedded spacer measuring efficiency, accuracy and electrochemical stability assessment

In order to ensure that the RTD embedded PLA spacer is electrochemically inert and will not introduce side reactions during LIB operation, the spacer was submerged in LIB electrolyte (1 M LiPF_6_ EC/DEC (Sigma-Aldrich)) for 5 days. A FTIR spectrum of the electrolyte was acquired and compared with the spectrum of the pristine electrolyte. For temperature measurement efficiency and accuracy evaluation, the RTD embedded spacer was firstly applied on a PID controlled hot stage, which was preset to various temperatures ranging from 30 °C to 70 °C. The spacer was mounted onto the hot stage with a pressure of 1500 psi (same pressure applied in cell crimping), while temperature readings from the RTD were recorded for 3 mins. Then, the CR2032 coin cell with RTD embedded spacer was cycled under vibration loading condition, as described in NAVSEA 9310^47^ and depicted in Fig. 3a. Four electrochemical cycles were completed and the electrochemical performance of the cell was analyzed.

### Short circuit testing with RTD embedded coin cell

In the short circuit test, a 15 mΩ shunt resistor was used for external shorting, and the total resistance of the external circuit was determined to be 19.8 mΩ. The low resistance external circuit generated a “hard” short circuit condition per NAVSEA 9310^47^. Four groups of RTD embedded LIBs with LCO cathode diameters of 9.5 mm, 12.5 mm, 14.9 mm, and 15.6 mm were prepared (LCO loading 14.53 mg/cm^2^) and analyzed in the short circuit test. Before testing, the cells were cycled between 3.0 V and 4.3 V for cathode electrolyte interface (CEI) formation^23^, which would decompose during the short circuit test and introduce further exothermic reaction^48^. The cells were then fully charged and shorted for 24 hours according to NAVSEA 9310^47^. Two RTDs were used for electrode and battery surface temperature measurement: one embedded on the spacer (internal RTD) and one attached on the battery surface (external RTD).

## Supplementary information

Supplementary Information

## Data Availability

The datasets generated during and analyzed during the current study are available from the corresponding author on reasonable request.
