# Supplementary material for: Lithium-ion Battery Thermal Safety by Early Internal Detection, Prediction and Prevention
Source: Sci Rep. 2019 Sep 13;9:13255. doi: 10.1038/s41598-019-49616-w (PMC6744460; doi:10.1038/s41598-019-49616-w)
Supplement: Supplementary file 1 — Supplementary Information [file 41598_2019_49616_MOESM1_ESM.pdf]

Lithium-ion Battery Thermal Safety by Early Internal Detection, Prediction and Prevention

Bing Li [1], Mihit H. Parekh [2], Ryan A. Adams [2], Thomas E. Adams [3], Corey Love [4], Vilas G. Pol [2,*], Vikas Tomar [1,*]

[1] School of Aeronautics and Astronautics, Purdue University, West Lafayette, IN 47907, USA

[2] Davidson School of Chemical Engineering, Purdue University, West Lafayette, IN 47907, USA

[3] Naval Surface Warfare Center, Crane Division, Crane, IN 47522, USA

[4] Naval Research Laboratory, Washington, DC 20375, USA

[*] Corresponding Authors, email: vpol@purdue.edu; tomar@purdue.edu

Supplementary Information

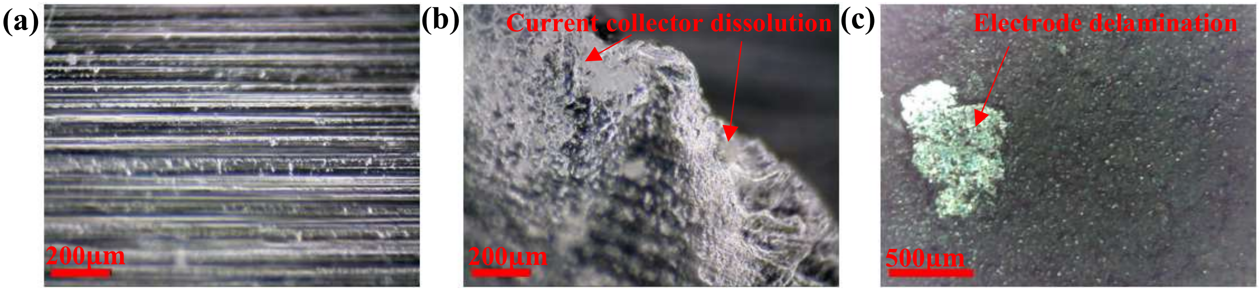

Supplementary Figure S1. (a): Pristine electrode current collector, (b): Current collector dissolution from short circuit, and (c): Electrode delamination from short circuit

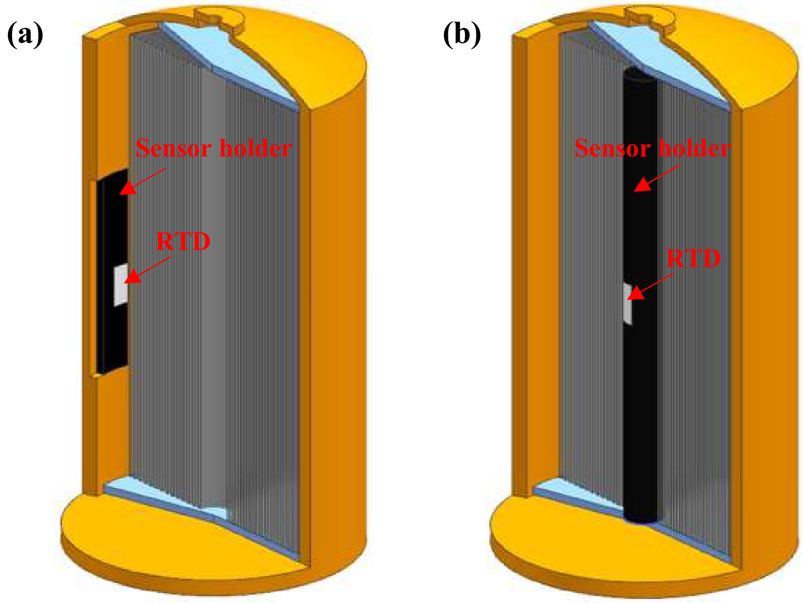

Supplementary Figure S2. Internal RTD in 18650 cell. (a): Application on battery case, and (b): Application at the core of jelly roll
